# SIRPα on Mouse B1 Cells Restricts Lymphoid Tissue Migration and Natural Antibody Production

**DOI:** 10.3389/fimmu.2020.570963

**Published:** 2020-10-09

**Authors:** Katka Franke, Saravanan Y. Pillai, Mark Hoogenboezem, Marion J. J. Gijbels, Hanke L. Matlung, Judy Geissler, Hugo Olsman, Chantal Pottgens, Patrick J. van Gorp, Maria Ozsvar-Kozma, Yasuyuki Saito, Takashi Matozaki, Taco W. Kuijpers, Rudi W. Hendriks, Georg Kraal, Christoph J. Binder, Menno P. J. de Winther, Timo K. van den Berg

**Affiliations:** ^1^ Sanquin Research and Landsteiner Laboratory, Department of Blood Cell Research, Amsterdam UMC, University of Amsterdam, Amsterdam, Netherlands; ^2^ Department of Pulmonary Medicine, Erasmus MC, Rotterdam, Netherlands; ^3^ Sanquin Research and Landsteiner Laboratory, Department of Plasma Protein, Amsterdam UMC, University of Amsterdam, Amsterdam, Netherlands; ^4^ Department of Medical Biochemistry, Experimental Vascular Biology, Amsterdam UMC, University of Amsterdam, Amsterdam, Netherlands; ^5^ Department of Pathology, CARIM, Cardiovascular Research Institute Maastricht, GROW-School for Oncology and Developmental Biology, Maastricht University, Maastricht, Netherlands; ^6^ Department of Laboratory Diagnostics, Medical University of Vienna, Vienna, Austria; ^7^ Division of Molecular and Cellular Signaling, Department of Biochemistry and Molecular Biology, Kobe University Graduate School of Medicine, Kobe, Japan; ^8^ Department of Pediatric Hematology, Immunology and Infectious Disease, Emma Children’s Hospital, Academic Medical Center, University of Amsterdam, Amsterdam, Netherlands; ^9^ Department of Molecular Cell Biology and Immunology, Amsterdam UMC, Vrije Universiteit Amsterdam, Amsterdam Infection and Immunity Institute, Amsterdam, Netherlands; ^10^ Institute for Cardiovascular Prevention (IPEK), Munich, Germany

**Keywords:** B1 cells, natural antibodies, atherosclerosis, immune checkpoint, inhibitory receptor, SIRPα, CD47, CD11b/CD18-integrin

## Abstract

The inhibitory immunoreceptor SIRPα is expressed on myeloid and neuronal cells and interacts with the broadly expressed CD47. CD47-SIRPα interactions form an innate immune checkpoint and its targeting has shown promising results in cancer patients. Here, we report expression of SIRPα on B1 lymphocytes, a subpopulation of murine B cells responsible for the production of natural antibodies. Mice defective in SIRPα signaling (SIRPα^ΔCYT^ mice) displayed an enhanced CD11b/CD18 integrin-dependent B1 cell migration from the peritoneal cavity to the spleen, local B1 cell accumulation, and enhanced circulating natural antibody levels, which was further amplified upon immunization with T-independent type 2 antigen. As natural antibodies are atheroprotective, we investigated the involvement of SIRPα signaling in atherosclerosis development. Bone marrow (SIRPα^ΔCYT^>LDLR^−/−^) chimaeric mice developed reduced atherosclerosis accompanied by increased natural antibody production. Collectively, our data identify SIRPα as a unique B1 cell inhibitory receptor acting to control B1 cell migration, and imply SIRPα as a potential therapeutic target in atherosclerosis.

## Introduction

Signal regulatory protein alpha (SIRPα) is an inhibitory immunoreceptor known to be expressed on myeloid and neuronal cells. SIRPα interacts with the broadly expressed cell surface ligand CD47 present on most cells in the body, including both hematopoietic and non-hematopoietic cells (1). Binding of CD47 to SIRPα generates intracellular inhibitory signals *via* immunoreceptor tyrosine-based inhibitory motifs (ITIM) in the cytoplasmic tail of SIRPα. Upon phosphorylation the SIRPα ITIM act to recruit and activate the tyrosine phosphatases SHP-1 and/or SHP-2, which inhibit tyrosine-phosphorylation-dependent signaling events and the resultant downstream cellular effector functions, including, e.g., phagocytosis (1). As such, the CD47-SIRPα axis forms an important innate immune checkpoint, with CD47 acting as so-called “don’t-eat-me” signal, which prevents the engulfment of healthy cells by myeloid cells (2). However, aberrant cells, such as cancer cells, may also exploit this pathway by (over)expressing CD47 and thus escaping immune-mediated destruction. Therapeutic targeting of the CD47-SIRPα checkpoint has been most intensively explored in the context of cancer. In fact, recent first in-human studies of agents interfering with this pathway demonstrate a favorable safety profile and promising therapeutic potential (3).

Based on their functions, anatomical location and phenotypical properties B lymphocytes can be divided into conventional B cells, also known as B2 cells, representing the majority of B cells, and into a smaller population of unconventional B1 cells. In mice, B1 cells are produced in the fetal liver before birth and afterward reside mainly in the pleural and peritoneal cavities where they are maintained by self-renewal (4). In addition, small proportions (<1%), but significant numbers, of these cells can be found in spleen and bone marrow (4–6). B1 cells residing in body cavities have a limited capacity to produce natural antibodies. However, after stimulation, by, e.g., LPS or viral infection, they migrate to the secondary lymphoid tissues, including the spleen, where they differentiate into plasma cells forming the major systemic source of natural antibodies (7, 8). This conditional migration is governed by the CD11b/CD18 integrin (7, 9). B1 cells that have arrived to the spleen gradually lose expression of CD11b/CD18 integrin, with hardly detectable levels after 6 days (9). Peritoneal B1 cells represent about 35%–70% of all CD19^+^ cells present in the peritoneal cavity and can be further divided into B1a (CD19^+^CD11b^+^CD5^+^) and B1b (CD19^+^CD11b^+^CD5^−^) cells (4). Unlike B2 cells, B1 cells in the spleen constitutively secrete natural antibodies, which are IgM antibodies commonly targeting, e.g., phospholipid and polysaccharide antigens, such as phosphorylcholine, phosphatidylcholine and lipopolysaccharide (4). Notably, a large part of the natural IgM antibodies is directed against epitopes created through lipid peroxidation (so called oxidation-specific epitopes, OSE), expressed amongst others on apoptotic cells and modified lipoproteins (10). Protective effects of natural antibodies against oxidized lipids have been well established in atherosclerosis (11–14), a chronic inflammatory disease characterized by accumulation of modified (oxidized) lipids in big and medium sized arteries (15). The atheroprotective capacity of IgM antibodies is explained by their binding to oxLDL, thereby preventing oxLDL uptake by macrophages, which as a consequence reduces foam cell formation and lesion development (11, 16). Additionally, natural antibodies are produced to promote clearance of apoptotic cells, which carry the same OSE as oxLDL (14).

It is known that B1 cell responses are restricted by different inhibitory immunoreceptors expressed on these cells, including, e.g., CD5 (17), CD22 (18), Fc gamma receptor IIb (FcγRIIb) (19, 20), and Siglec-G (21, 22). CD5 has been strongly linked to inhibition of BCR signaling, which prevents unwanted self-reactivity of B1 cells (23). B1 cells from mice lacking Siglec-G show a dramatic increase in Ca^2+^ flux upon anti-IgM treatment (22) and increased natural antibody production (24), also suggesting a role of Siglec-G in BCR signaling. All these receptors commonly exhibit their inhibitory functions through intracellular immunoreceptor tyrosine-based inhibitory motifs (ITIM), which upon tyrosine phosphorylation recruit and activate the cytosolic tyrosine phosphatases SHP-1 and/or SHP-2. In the case of FcγRIIb, the inositol phosphatases SHIP-1 and/or SHIP-2 play a prominent role as mediators of inhibitory signaling (25).

Here, we describe another inhibitory receptor, SIRPα, which is expressed on B1 cells in mice. We demonstrate that, in contrast to other currently known inhibitory receptors, SIRPα on B1 cells negatively regulates their migration, B1 cell numbers in the spleen, and systemic natural antibody production, without directly affecting B1 cell activation. Mice lacking the cytoplasmic tail of SIRPα (SIRPα^ΔCYT^ mice) in their hematopoietic compartment are protected against atherosclerosis with increased natural antibody levels against oxidized lipids. This identifies SIRPα as a novel immunoinhibitory receptor on B1 cells with unique regulatory functions and potential for therapeutic targeting in atherosclerosis.

## Materials and Methods

### Mice

SIRPα^−/−^ mice maintained on a C57BL/6 background have been described and were maintained in the Institute for Experimental Animals at Kobe University Graduate School of Medicine under specific-pathogen free conditions (26). C57BL/6 mice with a targeted deletion of the SIRPα cytoplasmic region have been described previously (27, 28). The mice that were originally generated onto the 129/Sv background were backcrossed onto C57BL/6 mice for at least 13 generations. Wild-type (wt) C57BL/6 mice of the same genetic background were maintained under specific pathogen-free conditions together with the SIRPα^ΔCYT^ mice in the breeding facility of The Netherlands Cancer Institute, Amsterdam, The Netherlands or the VU Medical Center, Amsterdam, The Netherlands. Unless indicated otherwise littermates from heterozygous breedings were used for both wild type and mutant mice. Bone marrow was isolated and used for transplantation at the animal facility of Maastricht University, Maastricht, The Netherlands. Animals were housed in ventilated cages and treated according to European Commission guidelines. They were euthanized using combination of isofluran and CO_2_. All animal experiments were approved by the Animal Welfare Committee of the VU Medical Center Amsterdam, The Netherlands, Maastricht University, Maastricht, The Netherlands, and The Netherlands Cancer Institute, Amsterdam, The Netherlands. LDLR^−/−^ mice on C57BL/6J background were obtained from Jackson Laboratory (Bar Harbor, ME, USA).

### Flow Cytometric Analysis of SIRPα Expression on Mouse B Cells

Mouse B cells were isolated from the peritoneal cavity by peritoneal lavage of 8–12 weeks old SIRPα^ΔCYT^ mice and age matched wt mice. Mice were sacrificed and immediately after that 5 ml of cold PBS containing 3% of fetal calf serum (FCS) and 3mM EDTA was injected into their peritoneal cavity. After gentle massage, cells were collected and used for analysis of SIRPα expression. Additionally, bone marrow and spleens of the same mice were isolated and blood samples were taken to analyze for expression of SIRPα. Fetal livers were isolated from mice of FVB background at E12. Single cell suspensions of splenocytes were prepared after the spleens were homogenized through 100µm filter (BD Biosciences, Bedford, MA, USA), lysed with lysis buffer and washed twice with cold PBS. For blood analysis whole blood was first spun down at 2,000 rpm at 4°C for 10 min and plasma was collected and stored at −80°C for later analysis of antibodies level. Erythrocytes were lysed using cold lysis buffer containing 155mM NH_4_Cl, 10mM KHCO_3_, and 0.1mM EDTA (ethylene diamine tetra acetic acid), pH 7.4. For flow cytometry analysis first Fc receptors were blocked using α-CD16/CD32 antibody (clone 2.4G2, BD Biosciences, Bedford, MA, USA). The cells were subsequently washed and stained for following surface markers with directly conjugated antibodies against CD19/B220 (PerCP Cy5.5 or eFluor 450), CD11b (Alexa Fluor 488), CD5 (PE), IgM (PE), SIRPα (APC or PerCP 710) (all antibodies purchased from eBioscience, San Diego, CA, USA), and CD43 (APC Cy7, BioLegend, San Diego, CA, USA). Expression of proteins was measured using FACS Canto II HTS (BD Biosciences, Bedford, MA, USA) and analyzed using FlowJo software (FlowJo LLC, Ashland, OR, USA). To reliably detect SIRPα expression and separate it from autofluorescence, fluorescence minus one (FMO) control was applied, when cells were stained for all determinants except SIRPα (9).

### Quantitative RT-PCR to Determine SIRPα mRNA Expression

RNA was isolated from FACS sorted mouse B1a and B2 cells based on markers listed above with QIAamp RNA Blood mini kit according to manufacturer’s instructions (Qiagen, Venlo, The Netherlands). RNA was eluted with 30 μl H2O, to obtain as high as possible concentration of RNA. Total RNA was reverse transcribed using the III first-strand synthesis system for RT-PCR (Invitrogen, Breda, The Netherlands). In short, 8-µl RNA was primed with 2.5 µM oligo-dT primer which specifically targets mRNA and 0.5 mM dNTP for 5 min at 65°C. Reverse transcription was performed with 10 U/µl Superscript III in the presence of 5 mM MgCl_2_, 20 mM Tris-HCL, and 50 mM KCl, pH 8.4 (RT buffer), 2 U/µl RNAseOUT™, lacking DTT for reasons described before (29) for 50 min at 50°C. After that, Superscript III was inactivated by incubation for 5 min at 85°C, followed by chilling on ice. Immediately thereafter, 2 U RNase H was added and incubated at 37°C for 20 min. Subsequently cDNA was stored at −20°C until further use. Amplification by PCR was performed on a LightCycler instrument (Roche, Almere, The Netherlands), with software version 3.5. The reaction was performed with Lightcycler FastStart DNA Master^PLUS^ SYBR Green I (Roche, Almere, The Netherlands). The annealing temperature used for all primers was 60°C. The reaction mix consisted of 4 µl of cDNA, 1 µM of each primer combination and 4 µl of Lightcycler FastStart DNA Master^PLUS^ SYBR Green I (Roche) in a total volume of 20 µl. After an incubation step for 10 min at 95°C, the template was amplified for 45 cycles at 95°C, annealing of the primers was performed at 60°C for 30 s, followed by extension at 72°C for 15 s. At the end of the 45 cycles, a melting curve was generated to determine the unique features of the DNA amplified. cDNA of control wt animals was used as a standard curve with a serial 10-fold dilution. Musculus Ubiquitin C was used as a reference gene. The product was sequenced by Big-dye Terminator Sequencing and ABI Prism software (Applied Biosystems, Foster City, USA). The sequence obtained was verified with BLAST (http://www.ncbi.nlm.nih.gov/BLAST/) to determine specificity. Primer sequences are available upon request.

### Binding of Phosphatidylcholine by Primary Mouse B Cells

B cells were isolated and labeled with antibodies against surface CD5 and B220 as described above along with fluorescein-labeled phosphatidylcholine (PtC) liposomes (DOPC/CHOL 55:45, Formumax Scientific Inc.). The cells were incubated on ice for 20 min followed by two washing steps, then cells were analyzed using an LSRII flow cytometer (BD Biosciences, Bedford, MA, USA) for binding of phosphatidylcholine and data were processed with FlowJo software (FlowJo LLC, Ashland, OR, USA).

### Intracellular Calcium Mobilization Measurement in Primary Mouse B1a Cells

B cells were isolated from the peritoneal cavity of 8–12 weeks old SIRPα^ΔCYT^ mice and aged matched wt mice using peritoneal lavage as described above. First, Fc receptors were blocked using α-CD16/CD32 antibody (clone 2.4G2, BD Biosciences, Bedford, MA, USA). The cells were then stained with directly labeled antibody against CD5 (APC) and B220 (APC Cy7, both BD Biosciences, Bedford, MA, USA) allowing identification of B1a cells. Calcium flux was determined as described before by flow cytometric determination (30). Briefly, intracellular fluxes of Ca^2+^ were measured using Fluo-3-AM and Fura Red-AM fluorogenic probes (Life Technologies, Carlsbad, CA, USA). The cells were incubated with 5μ Fluo3-AM and 5μM Fura Red-AM in loading buffer (Hank’s balanced salt solution medium supplemented with 10 mM HEPES and 5% fetal calf serum) at 30°C for 30 min in the dark. Cells were then washed and resuspended in buffer (Hank’s balanced salt solution medium with 10 mM HEPES, 5% fetal calf serum and 1 mM CaCl2) at room temperature. Cells were warmed to 37 °C for 5 min before acquisition of events. BCR-mediated Ca2^+^ mobilization was measured for 60s after the cells were stimulated either with 10 μg/ml F(ab′)2 of polyclonal goat anti-mouse IgM (Jackson ImmunoResearch, West Grove, PA, USA) or 0.5mM phosphatidylcholine (PtC) (F60103F-F, FormuMax USA). At the end of each Ca^2+^ measurement, cells were treated with ionomycine (Life Technologies, Eugene, OR, USA) as a positive control for calcium signaling. Data were acquired on an LSRII flow cytometer (BD Biosciences, Bedford, MA, USA) and data analysis was performed with the use of FlowJo software (FlowJo LLC, Ashland, OR, USA).

### Immunization of Mice With DNP-Ficoll

For B1-specific immunization intraperitoneal injection of TI-2 antigen di-nitro phenyl (DNP)-Ficoll was used as described originally in (31). Briefly, mice were injected with 50 μg of DNP-Ficoll in 200-μl PBS solution or with 200 μl of PBS only as control. After 7 days animals were sacrificed and their blood was collected, plasma was harvested and stored at −80°C before analysis of IgM and IgG3 antibodies by ELISA.

### Measurement of IgM and IgG With ELISA

Plasma levels of IgM antibodies against several OSE were determined by chemiluminescent ELISA (32). Dilutions of 1:100 [anti-phosphocholine (PC)-BSA IgM and all IgG antibodies], 1:500 [E06/T15id+ IgM, anti-malondialdehyde (MDA-)LDL IgM, anti-Cu-OxLDL IgM], and 1:20.000 (total IgM) were used. Supernatants of peritoneal B1 cell cultures or plasma of mice were serially diluted to determine IgM production after 48 h of stimulation or IgM/IgG3 against DNP-Ficoll after 7-days immunization as previously described (33). Briefly, supernatants were measured by sandwich ELISA, using unlabeled for coating and peroxidase‐labeled anti‐mouse IgM/IgG antibody (total, or DNP-Ficoll specific, Southern Biotechnology, Birmingham, AL, USA) for detection, and azino‐bis‐ethylbenz‐thiazoline sulfonic acid was used as the substrate. Antibody concentrations were calculated by using purified mouse IgM protein (IgM DNP-Ficoll and IgG3 DNP-Ficoll PMP52, Serotec, UK) as a standard.

### Proliferation of B1 Cells

B cells were isolated from the peritoneal cavity of either wt or SIRPα^ΔCYT^ mice and either left unstimulated or incubated with various stimuli for 48h after labeling with CFSE dye. Dilution of the dye after cell division was determined by flow cytometry on B1a cells (gated for CD19+, CD5+, CD11b+ lymphocytes) and percentage of proliferating cells was calculated.

### Stimulation of Peritoneal B1 Cell

Peritoneal B1a cells were obtained through negative magnetic-activated cell separation with a cocktail of antibodies depleting other than B1a cells achieving more than 90% purity in isolation (Miltenyi Biotec B.V., Utrecht, The Netherlands). B1a cells were subsequently counted and plated in IMDM medium (Invitrogen, Eugene, OR, USA) supplemented with 10% fetal calf-serum (FCS; Bodinco, Alkmaar, The Netherlands, 100 U/ml of penicillin, 100 mg/ml of streptomycin, and 2 mM L-glutamine (all Gibco Invitrogen, Breda, The Netherlands), and beta-mercapthoethanol (3.57 × 10^–4^M; Millipore, Amsterdam, The Netherlands). Cells were plated in 96-well plate in density of 1 × 10^6^/ml in 200 μl of medium and cultured at 37°C and 5% CO2 for 48h in presence of 5 μg of lipopolysaccharide (LPS, isolated from E. coli strain 055:B5, Sigma, St. Louis, MO, USA; LBP from R&D Systems, Abingdon, UK), isotype control (rat IgG2b, eBioscience, San Diego, CA, USA), anti-CD11b antibody (functional grade, eBioscience, San Diego, CA, USA), or anti-CD11a (functional grade, eBioscience, San Diego, CA, USA) in final concentration 10 μg/ml. After that supernatant was collected and stored at −80°C before measurement of IgM antibody by ELISA. Cells were harvested and processed for analysis by flow cytometry and imaging cytometry.

### Image Stream Analysis of Aggregate Formation

LPS-stimulated B1a cells were stained with following antibodies: CD19 (PerCP Cy5.5), CD11b (Alexa Fluor 488), CD5 (PE), SIRPα (APC), (all antibodies purchased from eBioscience, San Diego, CA, USA) and analyzed imaging cytometry to detect formation of aggregates (Image Stream, (Image Stream, Amnis, EMD, Millipore, Seattle, WA, USA) with gating strategy as follows: all events were divided based on their size into single cells (1 cell); doublets (2 cells); doublets and small aggregates (2–3 cells); big aggregates (3–4 cells); and large aggregates (>4 cells). Analysis of data was performed using analysis software IDEAS (Amnis Corporation, Seattle, WA, USA) and depicted as percentage of all gated events.

### Adoptive Transfer of Peritoneal B1 Cells

Either wt or SIRPα^ΔCYT^ animals were used as donors of peritoneal B1 cells for adoptive transfer. Cells were harvested by peritoneal lavage as described above. The cells were left in IMDM medium supplemented with 10% FCS (Bodinco, Alkmaar, The Netherlands, 100 U/ml of penicillin, 100 mg/ml of streptomycin, and 2 mM L-glutamine (all Gibco Invitrogen, Breda, The Netherlands) to rest for 30 min. After that the easy to detach and floating cells (excluding adherent peritoneal macrophages) were first incubated with Fc receptor blocking antibody (anti-CD16/CD32) and after washing incubated with either isotype control (rat IgG2b, eBioscience, San Diego, CA, USA) or anti-CD11b antibody (functional grade, eBioscience, San Diego, CA, USA) in concentration of 10 μg/ml for 30 min. Antibodies were washed away and wt and SIRPα^ΔCYT^ cells were labeled with membrane dye DiD and DiO, respectively (or vice versa, to exclude effect of the dye on cell properties). Cells were washed multiple times and mixed in 50:50 ratio based on a cell count. The actual ratio was additionally determined by analyzing a small sample of pooled cells on flow cytometer allowing later normalization of the cell input. Cells were then injected into the peritoneal cavity of either wt or SIRPα^ΔCYT^ recipient mice, left resting for 1 h, and followed by either 200-μl injection of PBS (control) or 10 μg of LPS in 200-μl PBS intraperitoneally to induce migration of B1 cells from the peritoneal cavity (7, 33). Peritoneal lavage of recipient mice was performed 3h after PBS/LPS injection. Lavage composition was analyzed by flow cytometry in CD19+ single cell population and percentage of cells with distinct membrane label was calculated. Percentage of cells was normalized for input of pooled cells as indicated above.

### Bone Marrow Transplantation

One week before transplantation, female LDLR^−/−^ mice were housed in filter top cages with neomycin (100 mg/L; Gibco, Breda, The Netherlands) and polymyxin B sulphate (66104 U/L; Gibco Breda, The Netherlands) in their acidified drinking water. The animals received 6 Gy of total body irradiation twice on consecutive days. Bone marrow isolated from SIRPα^ΔCYT^ and wt mice was injected intravenously to rescue the hematopoietic system of the irradiated mice as described previously (34). Briefly, one week before transplantation, female LDLR−/− mice were housed in filter-top cages and provided with acidified water containing neomycin (100mg/l; GIBCO, Breda, The Netherlands) and polymyxin B sulfate (6 × 104 U/l; GIBCO). The animals received 2 × 6 Gy total body irradiation on two consecutive days. On the second day, bone marrow was isolated from 6 SIRPα^ΔCYT^ and 6 wt littermates, and 10^7^ cells/mouse were injected intravenously to rescue the hematopoietic system of the irradiated mice. Four weeks after the transplantation, mice were put on a high fat diet (0.15% cholesterol, 16% fat, Arie Blok, The Netherlands) for 10 weeks and level of chimerism was tested (reached 98.76% ± 0.73).

### Mouse Blood Parameters

Blood was withdrawn at the indicated times during high fat diet period and plasma lipid levels were enzymatically measured using ELISA (Sigma Aldrich, Zwijndrecht, The Netherlands).

### Atherosclerotic Lession Analysis

Transplanted animals were sacrificed and isolated hearts were cut perpendicularly to heart axis just below the atrial tips, as described before (35, 36). Briefly, tissue was frozen in tissue-tec (Shandon, Veldhoven, The Netherlands) with the base facing downward, and sectioning was performed toward the aortic valve area. Sections of 7 μm were collected, starting from where the atrioventricular valves were visible. Aortic lesion areas were quantified using serial cross-sections obtained every 42 μm, beginning at the start of the atrioventricular valves and spanning 250 μm. Serial cross sections were stained with toluidin blue and lesion areas were quantified using Adobe Photoshop software. Severity of lesions was scored as early, moderate and advanced, using criteria as described before (35, 36). In detail, early lesions were fatty streaks containing only foam cells; moderate (intermediate) lesions were characterized by the additional presence of a collagenous cap, and advanced lesions showed involvement of the media mostly accompanied by increased collagen content and necrosis of the plaque. Foam cell size within plaques was determined by dividing the size of an allocated foamy macrophage area by the number of macrophages.

### Immunohistochemical Staining

Atherosclerotic lesions from aortic roots were stained with various antibodies to identify neutrophils (NIMP directed against Ly6G, a gift from P. Heeringa), T cells (KT3, directed against CD3) and newly recruited macrophages (ER-MP58, a gift from P. Leenen) followed by detection with biotin labeled rabbit anti-rat antibody and staining with ABC kit (Vector Labs, Burlingame, CA).

### Statistical Analysis

Statistical analysis was performed using GraphPad Prism version 8.02 (GraphPad Software, San Diego, CA, USA). Data were evaluated by two-tailed student t-test if two columns were compared. If more columns were compared, one-way ANOVA followed by multiple comparison correction was applied.

## Results

### SIRPα Is Expressed on B1 Cells

**Figure 1:**
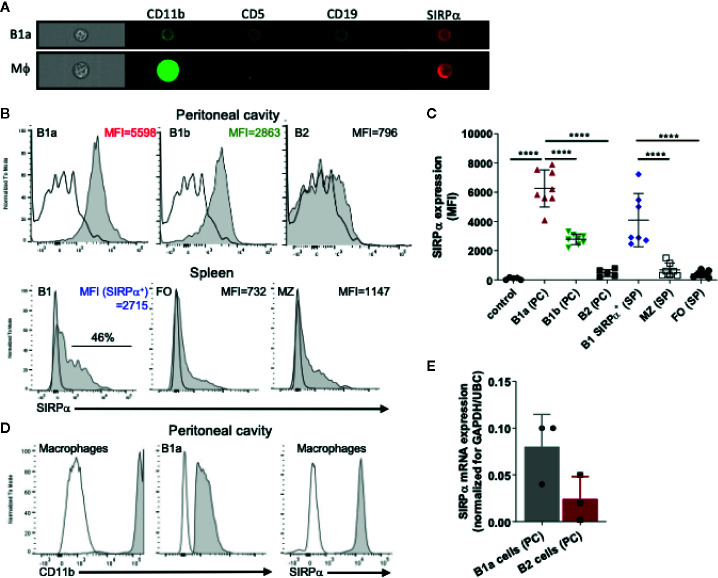
SIRPα is expressed by B1 cells. **(A)** Imaging flow cytometry visualizing expression of SIRPα on CD19+CD5+CD11b+ B1a cells and CD11b+CD19− macrophages. Representative histograms **(B)** and synopsis **(C)** of SIRPα surface expression on defined B cell subpopulations as determined by flow cytometry (MFI, Mean fluorescence intensity). Note that the most prominent expression occurs on peritoneal cavity (PC) B1a and B1b cells, and on a subset of the splenic (SP) B1, with little or no expression on marginal zone (MZ) and follicular (FO) B cells. **(D)** Macrophages cells from the peritoneal cavity show relatively high levels of expression of both CD11b and SIRPα. **(E)** SIRPα mRNA expression on FACS sorted peritoneal cavity B1a and B2 cells. Data are in **(C, E)** are presented as mean ± SEM and represent measurements of 5–8 and 3 individual mice, respectively. Statistical analysis was performed by one-way ANOVA with Dunnett’s multiple comparison corrrection, ****p < 0.0001.

The inhibitory immunoreceptor SIRPα is considered to be present selectively on neuronal cells as well as on myeloid cells in the hematopoietic compartment (1, 37). It is thought to be lacking from lymphoid cells, at least under steady state conditions (38). However, a more detailed evaluation of B cell subsets revealed SIRPα expression on all B1 cells in the peritoneal cavity (PC) and on a subpopulation of B cells in the spleen (SP) of mice (**Figures 1A–C** and **Supplementary Figures 1A, B**). In particular, by using specific markers identifying B1a cells (i.e., CD19^+^CD5^+^CD11b^+^) and B1b cells (i.e., CD19^+^CD5^−^CD11b^+^) we could clearly demonstrate surface SIRPα expression on both of these PC B1 lymphocyte populations. PC B2 cells show much lower if any SIRPα expression. In the spleen we could detect expression of SIRPα only on a subset of B220^+^/CD19^+^CD43^+^CD23^−^ B1 cells. Relatively low levels of SIRPα staining were found on marginal zone B cells (B220^+^/CD19^+^CD43^−^CD23^−^) and minimal detectable expression was found on B220^+^/CD19^+^CD23^+^CD43^−^ follicular B cells. Consistent with other studies (38), we could not observe any SIRPα expression on circulating B cells in mice and no expression on B2 cells from the bone marrow (**Supplementary Figure 1C**). However, we could detect SIRPα on fetal liver B220^+^/CD19^+^CD43^+^ B cells and B1 cells in the bone marrow (**Supplementary Figure 1C**). As a control staining was performed on B1 cells from the peritoneal cavity of mice deficient for SIRPα altogether (SIRPα^−/−^ mice, **Supplementary Figure 2**) indicating that staining observed on B1 cells in wild type mice can indeed be solely attributed to SIRPα expression on those cells. Staining on B1 cells from SIRPα^ΔCYT^ mice showed a slightly reduced overall surface expression as has been reported for other cells expressing SIRPα (data not shown). Compared with peritoneal macrophages, we found substantially lower levels of both CD11b and SIRPα on B1 cells, clearly discriminating B1 cells from myeloid cells (**Figure 1D**). Expression of SIRPα mRNA was confirmed by qRT-PCR on FACS sorted peritoneal B1a cells (CD19^+^CD5^+^CD11b^+^) (**Figure 1E**).

### SIRPα Limits Natural IgM Antibody Levels *In Vivo*

**Figure 2:**
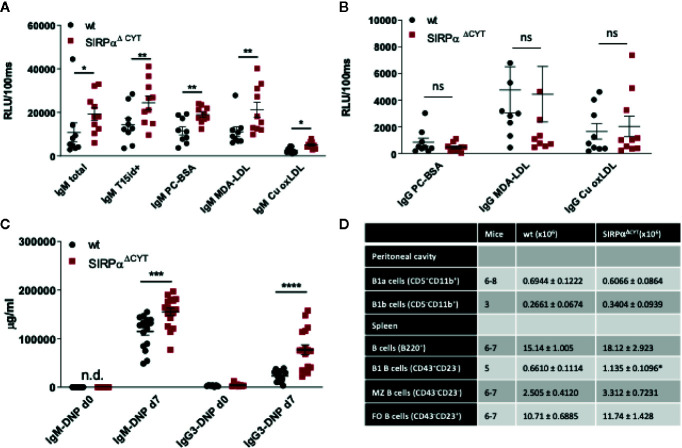
Loss of SIRPα signaling promotes B1 cell accumulation in the spleen and natural IgM antibody formation *in vivo*. Defective SIRPα signaling in mice lacking the SIRPα cytoplasmic tail (SIRPα^ΔCYT^) results in increased plasma levels of natural IgM **(A)** but not IgG **(B)** antibodies directed against the indicated oxidation-specific epitopes under steady-state conditions; wt, wild-type mice; RLU/100 ms, Relative Luminescence per 100 ms. **(C)** Immunization with the haptenated TI-2 antigen DNP-Ficoll triggers increased production of both IgM and IgG3 antibodies against DNP in SIRPα^ΔCYT^ mice. Data are presented as mean ± SEM and are representative of 9-10 **(A)**, 8 **(B)**, 17 **(C)** individual mice. **(D)** B cell numbers in peritoneal cavity and spleen of wt and SIRPα^ΔCYT^ mice. Absolute number of different B cell populations was determined in the peritoneal cavity and the spleen of young adult mice (8–12 weeks) under steady state conditions. For further details see **Supplementary Figures 2A–G**. Statistical analysis was performed by unpaired Student t-test, corrected for multiple comparisons with Holm-Sedak method where applicable, *p < 0.05; **p < 0.01, ***p < 0.001, ****p < 0.0001.; ns, non-significant; n.d., not detectable.

Because SIRPα, like Siglec-G, is also a typical inhibitory immunoreceptor with cytoplasmic ITIM motifs signaling through SHP-1 and/or SHP-2, we tested whether the lack of SIRPα signaling would affect natural antibody generation as well. As can be seen in **Figure 2A** there was a prominent (~2-fold) enhancement in the plasma levels of total and OSE-reactive natural IgM antibodies in mice lacking the cytoplasmic tail of SIRPα (SIRPα^ΔCYT^ mice). This occurred for all OSE tested, including the so-called T15 epitope that defines PC-reactive EO6 type anti-OxLDL IgM antibodies with anti-atherogenic potential *in vivo* (11, 39), as well as phosphocholine (PC) and those against *ex vivo* oxidized LDL (i.e., MDA-LDL and Cu-OxLDL). Consistent with a specific B1 cell phenotype and a selective regulation of natural IgM levels the corresponding IgG levels were not altered (**Figure 2B**). When SIRPα^ΔCYT^ mice were immunized with a typical T-cell independent type 2 (TI-2) antigen, DNP-Ficoll, we observed robust and enhanced DNP-specific IgM and IgG3 immune responses (**Figure 2C**). Of interest, no enhanced antibody responses have been observed in SIRPα^ΔCYT^ mice upon immunization with the thymus-dependent antigen TNP-KLH (Y. Kaneko (Gunma University, Japan), personal communication). These results indicate that SIRPα signaling regulates natural antibody production as well as the response of B1 cells to antigenic stimulation.

### SIRPα Increases Splenic B1 Cell Numbers Without Affecting B Cell Receptor Function

**Figure 3:**
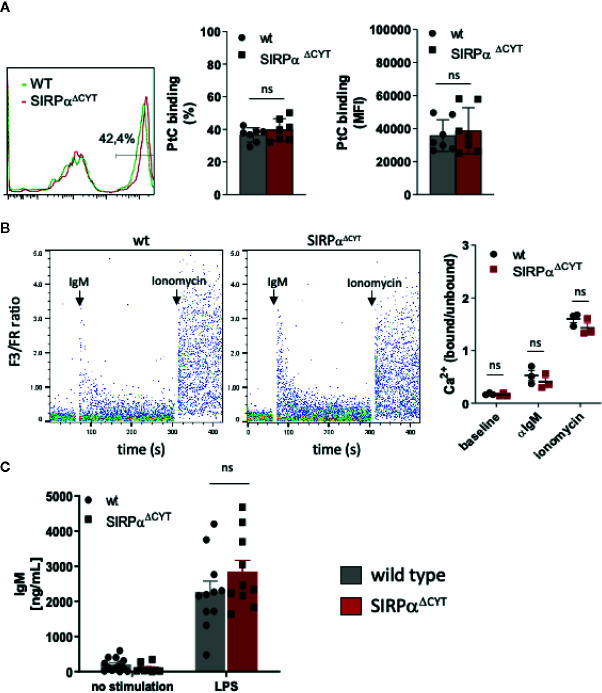
Loss of SIRPα signaling does not have a generalized effect on B1 cell activation. **(A)** Comparable frequency (FACS plot of representative example, left panel; % positivity, middle panel) and magnitude (MFI, right panel) of phosphatidylcholine (PtC; a typical B1a antigen) antigen binding by wt and SIRPα^ΔCYT^ peritoneal cavity B1a cells (n = 7 mice/group). **(B)** Similar levels of B1a cell surface IgM signaling, as determined by intracellular Ca^2+^ mobilization, in wt and SIRPα^ΔCYT^ peritoneal cavity B1 cells triggered by anti-IgM antibodies; responses with ionomycin are shown as a positive control; left panels: representative examples of Ca^2+^-responses in wt and SIRPα^ΔCYT^ **(B)**; right panels: average values of B1a cells from n = 3 mice/group. **(C)** Comparable levels of LPS-stimulated IgM production in B1a cells isolated from SIRPα^ΔCYT^ and wt mice (n = 10–11 mice/group); ns, non-significant.

Next, we investigated whether the changes in B1-cell associated antibodies in SIRPα^ΔCYT^ mice could be related to changes in B1 cell numbers in the peritoneal cavity or the spleen of these mice (**Figure 2D**, **Supplementary Figures 3A–G**). We did not detect significant differences in proportions or the absolute numbers of peritoneal cavity B1a and B1b cells. In contrast, proportions and absolute numbers of B1 cells in the spleen of SIRPα^ΔCYT^ mice were significantly (~2-fold) increased compared to wt animals. There were no significant differences in total cell numbers or proportions of other splenic B cell subsets, indicating that the effects were specific for B1 cells. We next asked how SIRPα might contribute to the increase in splenic B cell numbers and natural antibody levels. One possibility was that SIRPα was controlling the activation and expansion of B1 cells. First, we tested whether antigen recognition by B1 cells might be altered, as a consequence of potential differences in BCR expression, but we observed no difference in, e.g., the binding of a typical B1a cell antigen phosphatidylcholine (PtC) (40) to B1a cells (**Figure 3A** and **Supplementary Figure 3H**). Next, we explored potential differences in B1a cell activation capacity. This included measuring Ca^2+^ flux upon cross-linking of BCR (**Figure 3B**), and monitoring sorted PC B1a cell IgM secretion upon stimulation with LPS *in vitro* (**Figure 3C**), a potent and typical B1 cell activation stimulus (7). Both read-outs showed comparable activation capacity of wt and SIRPα^ΔCYT^ B1a cells. Finally, there were apparently no differences in B1a cell proliferation (**Supplementary Figure 4**). Taken together, these findings support the idea that SIRPα signaling controls splenic B1 cell accumulation and, likewise as a consequence, also the levels of naturally occurring antibodies, but this was apparently not linked to a generalized regulation of BCR- or TLR-mediated B1 cell activation.

### SIRPα Regulates CD11b/CD18 Integrin Function and B1 Cell Migratory Capacity

**Figure 4:**
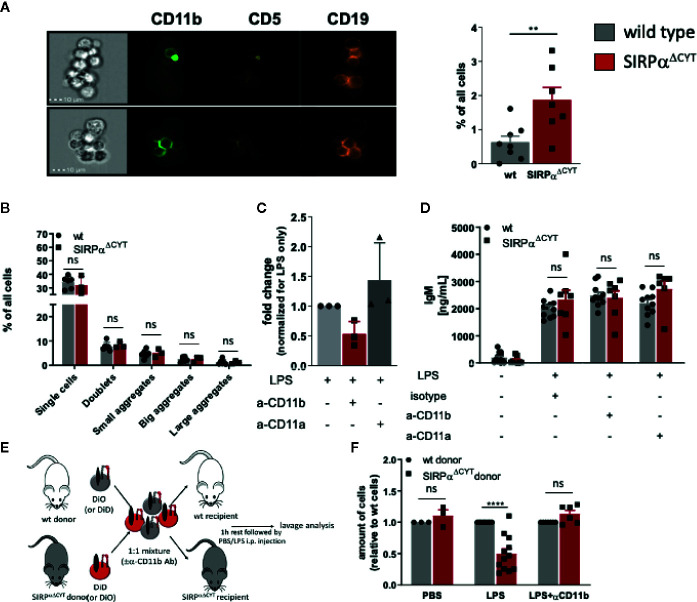
Loss of SIRPα signaling promotes LPS-induced CD11b/CD18 integrin-dependent B1 cell efflux from the peritoneal cavity. **(A, B)** Lack of SIRPα signaling promotes the formation of large aggregates in isolated SIRPα^ΔCYT^ peritoneal cavity **(A)** but not splenic **(B)** B1a cells (n = 6–8 mice/group). **(C)** Enhanced frequency of B1 cell large aggregates in SIRPα^ΔCYT^ peritoneal cavity B1a cells relative to wt cells is CD11b/CD18-integrin-dependent as it is reduced by blocking anti-CD11b, but not anti-CD11a antibodies. **(D)** Blockade of CD11b/CD18 or CD11a/CD18 integrins has no effect on IgM antibody production by peritoneal cavity B1 cells upon LPS stimulation. **(E)** Experimental design of egress of adoptively transferred mixed SIRPα^ΔCYT^/wt B1 cells from the peritoneal cavity. **(F)** Evaluation of adoptively transferred mixed SIRPα^ΔCYT^/wt B1 cells shows increased efflux of SIRPα^ΔCYT^ B1 cells relative to wt from the peritoneal cavity upon LPS stimulation and this enhanced egress is CD11b/CD18 dependent (n = 3–12 mice/group). Similar data were obtained for SIRPα^ΔCYT^ recipients (not shown). Data are presented as mean ± SEM. Statistical analysis was performed by unpaired Student t-test, corrected for multiple comparisons with Holm-Sedak method where applicable, **p < 0.01, ****p < 0.0001; ns, non-significant.

Notably, when analyzing LPS-stimulated B1 cells by flow cytometry, we observed the presence of cell clusters in the cultures that appeared larger for SIRPα^ΔCYT^ B1a cells relative to their wt counterparts (**Supplementary Figure 5A**). This prompted us to visualize and quantify this aggregate formation of B1a cells by imaging flow cytometry, which indeed consistently demonstrated a substantially increased proportion of large aggregates (i.e., consisting of more than 4 cells) in SIRPα^ΔCYT^ peritoneal B1a cells as compared to wt B1a cells after LPS stimulation (**Figure 4A** and **Supplementary Figures 5B, C**). In contrary, we could not observe comparable aggregates when sorted splenic B1 cells were cultured in a similar manner (**Figure 4B**). Interestingly, such doublets and large aggregates specific for CD11b^+^ B1 cells have been previously reported by Ghosn et al. and their formation seems to be dependent on CD11b (9). Furthermore, SIRPα inhibitory signaling has been previously linked to integrin function in other cells (28). We therefore hypothesized that SIRPα may serve as a negative regulator of CD11b/CD18 integrin function in B1 cells. To test this, we stimulated sorted peritoneal B1a cells with LPS in the presence of a blocking anti-CD11b antibody. Clearly, the increased formation of large aggregates triggered by LPS in mice lacking SIRPα signaling could be partially prevented by blocking CD11b, but not by blocking CD11a (**Figure 4C**). Next, we asked whether B1 cell aggregate formation through CD11b/CD18 integrin could be a prerequisite for production of natural antibodies. We tested supernatants of B1a cells that were activated by LPS in the presence of blocking CD11b antibody or blocking CD11a antibody. It appeared that SIRPα^ΔCYT^ B1a cells have comparable antibody production as wt B1 cells *in vitro*, independently of CD11b or CD11a function (**Figure 4D**). Thus, whereas CD11b-mediated formation of large aggregates did not regulate natural antibody production *in vitro*, such B1 cell aggregate formation, which was promoted upon disruption of SIRPα signaling, nevertheless appeared a read-out for CD11b/CD18 activation. This suggested that SIRPα signaling was negatively regulating B1 cell integrin function. Of interest, Waffarn et al. have shown, that CD11b/CD18, unlike CD11a/CD18, is indispensable for migration of stimulated B1 cells from cavities to secondary lymphoid tissues where they mature into natural antibody producing plasma cells (8). This led us to propose that SIRPα could actually regulate CD11b/CD18 function during migration of B1 cells to the secondary lymphoid tissues, which would provide an explanation for the increased numbers of B1 cells in the spleens of SIRPα^ΔCYT^ mice. To directly test the effect of SIRPα signaling on the capacity of B1 cells to migrate from the peritoneal cavity we performed adoptive transfer experiments. To confirm integrin-dependence, B1 cells of both wt and SIRPα^ΔCYT^ donor mice were in parallel pre-incubated with blocking CD11b antibody, excessive amount of the antibody was removed, and then, the cells were labeled with unique membrane dyes, mixed in 1:1 ratio, and injected to either wt or SIRPα^ΔCYT^ recipients (**Figure 4E**). This set-up allowed us to selectively monitor, in individual animals, the effect of SIRPα and CD11b/CD18 on the migration of B1 cells from the peritoneal cavity to the spleen. As expected, B1 cells that lack inhibitory cytoplasmic tail of SIRPα showed increased efflux from the peritoneal cavity (**Figure 4F**). The enhanced exit of SIRPα^ΔCYT^ B1 cells was fully dependent on CD11b/CD18, as it could be completely inhibited by antibody blocking. Taken together, our data strongly suggest that CD11b/CD18 function in B1 cells is under negative control of SIRPα and that in absence of SIRPα signaling B1 cells have a higher propensity to leave the peritoneal cavity, thereby contributing to an accumulation of B1 cells in the spleen and an enhanced systemic production of natural antibodies.

### Lack of SIRPα Signaling Protects Mice From Atherosclerosis

**Figure 5:**
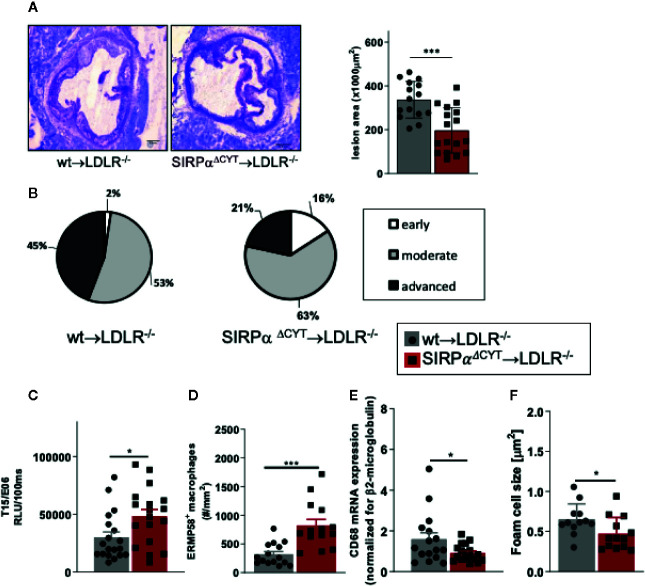
Loss of SIRPα signaling protects mice from atherosclerosis. **(A, B)** Bone marrow chimeras carrying dysfunctional SIRPα in their hematopoietic compartment are protected from atherosclerosis, showing **(A)** smaller and **(B)** less severe aortic lesions. **(C)** Atheroprotection in SIRPα^ΔCYT^>LDLR^−/−^ chimeras is associated with increased levels of oxLDL-targeting natural antibody T15/E06 in plasma. **(D–F)** Atherosclerotic lesions of SIRPα^ΔCYT^>LDLR^−/−^ chimeras contain more small macrophages **(D)**, less CD68+ macrophages **(E)**, and a smaller foam cell area **(F)** as compared to wt chimeras. Data are presented as mean ± SEM and are representative of 15-17 **(A, B)**, 18-19 **(C)**, and 12-16 **(D–F)** individual mice. Statistical analysis was performed by unpaired Student t-test used per variable, corrected for multiple comparisons with Holm-Sedak method, *p < 0.05; ***p < 0.001 or Chi-square test **(B)**, *p < 0.05.

In order to further establish the potential pathological/clinical relevance of the regulation of natural IgM antibody production by SIRPα *in vivo*, we decided to explore the role of SIRPα in atherosclerosis. Natural IgM antibodies produced by B1a cells have a well-established protective role in various diseases, and particularly in atherosclerosis, a feature which is apparently due to their capacity to neutralize oxLDL uptake and enhance apoptotic cell clearance by macrophages (11–13). To directly address the role of SIRPα in atherosclerosis in mice, we transplanted wt and SIRPα^ΔCYT^ bone marrow into atherosclerosis-prone LDLR^−/−^ recipient mice. This well-established atherosclerosis model includes the replacement of peritoneal B cell populations (including B1 cells) by the donor cells (11, 12, 21, 22, 41–43). Mice transplanted with wt or SIRPα^ΔCYT^ cells, and subjected to a high-fat diet, showed neither differences in weight, plasma cholesterol and triglyceride levels nor prominent changes in blood cell composition (**Supplementary Figures 6A–E**). However, when the atherosclerotic lesions of these mice were evaluated, it became apparent that mice transplanted with SIRPα^ΔCYT^ cells developed much smaller lesions (**Figure 5A**) with a substantially less severe phenotype (**Figure 5B**) compared to wt chimeras. Additionally, when the plasma of atherosclerotic mice was analyzed for the presence of antibodies against oxLDL, in mice transplanted with SIRPα^ΔCYT^ cells elevated levels of T15/E06 IgM (**Figure 5C**), an oxLDL neutralizing antibody, being particularly critical in the protection against atherosclerosis, were found. (11, 39, 44). Similar to the steady state situation, levels of IgG targeting OSE remained unaltered (**Supplementary Figure 6F**). A more detailed evaluation of the cellular composition of the lesions showed an increase in the number of newly recruited ERMP58+ myeloid cells (**Figure 5D**), which was associated with a significantly decreased mRNA for CD68^+^ macrophages (**Figure 5E**) and size of the area occupied by foam cells in mice transplanted with SIRPα^ΔCYT^ cells (**Figure 5F**), consistent with the proposed mechanism of IgM-mediated inhibition of foam cell formation (11, 16, 45). There were no significant differences observed in numbers of other immune cells known to be involved in pathogenesis of atherosclerosis, such as T-lymphocytes and neutrophils (**Supplementary Figures 6G, H**).

## Discussion

In this study, we provide evidence for the expression and functional relevance of the inhibitory receptor SIRPα on B1 cells in mice. Our results demonstrate that SIRPα is an inhibitory receptor on B1 cells that controls the numbers of splenic B1 cells, thereby most likely affecting systemic natural antibody levels. The increase in splenic B1 cell numbers in the absence of SIRPα signaling occurs most probably because of the absence of an inhibitory effect of on CD11b/CD18 integrin activation, which promotes the migration of these B1 cells from the peritoneal cavity to the spleen. We also show that a lack of inhibitory SIRPα signaling in atherosclerotic mice leads to selectively elevated plasma levels of oxLDL-neutralizing natural antibodies and propose that this directly contributes to the atheroprotective effect of SIRPα mutation. The latter is in agreement with the well-established regulatory role of such antibodies in atherosclerosis (46).

SIRPα is one of the most abundant inhibitory receptors on myeloid cells including neutrophils, monocytes, the majority of tissue macrophages and CD4^+^ dendritic cells, affecting a variety of cell functions in a primarily negative fashion (1, 2). SIRPα has, as yet, not been reported to be expressed by any B cells. Recently, SIRPα was also reported to be selectively expressed on a small subset of T lymphocytes, i.e., exhausted CD8+ memory T cells emerging after chronic viral infection (47). For a long time, the general assumption has been that SIRPα is, at least among hematopoietic cells, restricted to the myeloid lineage (37, 48). Most of the studies based the absence of SIRPα from lymphocytes on staining of blood cells in rodents (37, 38) while other, less accessible or more obscure subpopulations of lymphoid origin remained unexplored. We found expression of SIRPα exclusively on B1 cells in the peritoneal cavity and on a minor subset of B cells in the spleen. We also observed that the population of steady-state splenic B1 cells is roughly doubled in mice lacking SIRPα signaling. This increase in splenic B1 cells may well explain the (also ~2-fold) higher IgM plasma levels found in SIRPα^ΔCYT^ mice. Antigens with repetitive patterns, such as lipids and glycolipids, including self-antigens that are generated, e.g., upon oxidation or apoptosis can induce multivalent antigen crosslinking of specific BCR on B1 cells and induce TI-2 responses. Due to this self- and poly- reactivity of B1 cells, their functions have to be tightly regulated to avoid autoimmunity. Several inhibitory receptors that regulate various aspects of B1 cell function are already known to be instrumental in this. We have observed that lack of SIRPα on B1 cells has no measurable effect on calcium flux and IgM secretion, which may suggest that Siglec-G and CD5, which have previously shown to regulate these parameters, comprise the major regulators of BCR signaling in B1 cells (17). SIRPα^ΔCYT^ mice have moderately increased numbers of B1 cells only in the spleen with the peritoneal population virtually unaltered. Also, B1-associated antibody levels are elevated in SIRPα^ΔCYT^ mice, both at baseline and after TI-2 antigen exposure. Natural antibodies are predominantly produced by B1 cells in secondary lymphoid organs such as spleen (4) or specific (e.g., mediastinal) lymph nodes (8). Egress of B1 cells residing in the body cavities (peritoneal or pleural) into the secondary lymphoid organs has been demonstrated to depend on the CD11b/CD18 integrin (8). CD11b can function only in heterodimer with CD18 integrin, forming together CD11b/CD18 (also known as CR3, Mac-1, Integrin alpha M, or α_M_β_2_ integrin). B1 cells are known to express various integrin molecules, but B1 cells are the only B cells that express CD11b/CD18 integrin and until now no direct regulator of its function has been described. We show here that SIRPα negatively regulates capacity of B1 cells to exit the peritoneal cavity through CD11b/CD18 integrin, identifying this inhibitory immunoreceptor as the first *bona fide* regulator of B1 migratory function. It should be emphasized that we cannot formally attribute the *in vivo* phenotype of the SIRPα-mutant mice to B1 cell-intrinsic effects of SIRPα signaling, since also other cells, such as myeloid cells, in these mice have a defect in SIRPα signaling as well. Taken together, a picture emerges where different B1 cell functions appear to be regulated by distinct inhibitory receptors, with SIRPα more or less specifically controlling their migratory behavior, whereas others, e.g., Siglec-G and CD5 may control B1 cell activation in a more generalized fashion.

The homeostatic function of IgM antibodies has been well documented in atherosclerosis (11–13, 49). Mice lacking SIRPα signaling in the hematopoietic compartment showed increased plasma levels of T15/E06 IgM and show smaller and less severe atherosclerotic lesions. This is consistent with observations in Siglec G^−/−^ bone marrow chimeras, where the lack of inhibitory signaling by Siglec G led to increased OSE-specific natural IgM antibody levels and decreased atherosclerosis development (24). Increased plasma level of T15/E06 IgM is a very likely mechanism of atheroprotection in SIRPα^ΔCYT^ chimeras. However, transplantation of SIRPα^ΔCYT^ and wt bone marrow resulted in replacement of both myeloid and lymphoid lineage in the donor LDLR^−/−^ mice. As macrophages are important players in development of atherosclerosis we cannot exclude their contribution to the observed phenotype, especially since the SIRPα counter-receptor CD47 appears involved in pathogenesis of atherosclerosis through inhibition, e.g., macrophage efferocytosis (50). Furthermore, whether blocking antibodies targeting SIRPα, rather than CD47, would show similar effect still remains to be confirmed, also because the CD47 monoclonal antibody miap410 used in the study of Kojima (50) has prominent opsonizing capacity and a less convincing ability to actually block the CD47-SIRPα axis (51).

Importantly, it is well established that humans, like mice, have “natural” antibodies targeting, e.g., OSE, which are atheroprotective, and these even appear to have prognostic value for the development of cardiovascular disease (52). Based on our current findings in mice, the prediction would therefore be that the human natural antibody producing B cells would also express SIRPα and would consequently also be subject to regulation *via* the CD47-SIRPα axis. The problem is, however, that the B1 cell equivalent subset responsible for natural antibody production in humans has not been properly identified, and this is in fact a heavily debated issue in the B1 cell field. For instance, the reported identification of human B1 cells in blood defined as CD20^+^CD27^+^CD43^+^CD70^−^ (53) is quite controversial and has been challenged by several other studies suggesting that these cells are rather result of a technical artefact (54–56). Further studies are clearly needed to resolve this issue and to establish SIRPα expression and function on these putative human B1 cells.

Collectively, our data identify SIRPα as a novel B1 cell immune checkpoint, which functions to control B1 cell migration to lymphoid tissues and natural antibody generation. Our findings also imply SIRPα as a potential therapeutic target in atherosclerosis. The CD47-SIRPα innate immune checkpoint is currently extensively studied in the context of cancer immunotherapy (2, 57), with a number of different agents in preclinical and/or clinical development, and ~35 ongoing clinical trials, carving out a path for potential therapeutic targeting of the CD47-SIRPα axis also in other diseases, including cardiovascular disease.

## Author’s Note

This manuscript has been released as a preprint at BioRxiv, doi: https://doi.org/10.1101/2020.05.13.092494.

## Data Availability Statement

The raw data supporting the conclusions of this article will be made available by the authors, without undue reservation.

## Ethics Statement

The animal study was reviewed and approved by Animal Welfare Committees of the VU Medical Center Amsterdam, The Netherlands; Maastricht University, Maastricht, The Netherlands; and The Netherlands Cancer Institute, Amsterdam, The Netherlands.

## Author Contributions

KF, SP, MH, MG, HM, JG, HO, CP, PG, MO-K, and YS performed experiments and analyzed data. TM, TK, RH, GK, CB, MW, and TB provided reagents and facilities and designed research and/or evaluated data. KF and TB wrote the paper. All authors contributed to the article and approved the submitted version.

## Funding

This work was supported by NWO-TOP grant (#91208001) awarded to GK, MW, and TB.

## Conflict of Interest

The authors declare that the research was conducted in the absence of any commercial or financial relationships that could be construed as a potential conflict of interest.
