# Supplementary material for: SIRPα on Mouse B1 Cells Restricts Lymphoid Tissue Migration and Natural Antibody Production
Source: Front Immunol. 2020 Oct 9;11:570963. doi: 10.3389/fimmu.2020.570963 (PMC7581795; doi:10.3389/fimmu.2020.570963)
Supplement: Supplementary file 1 [file DataSheet_1.pdf]

Supplementary Fig. 1

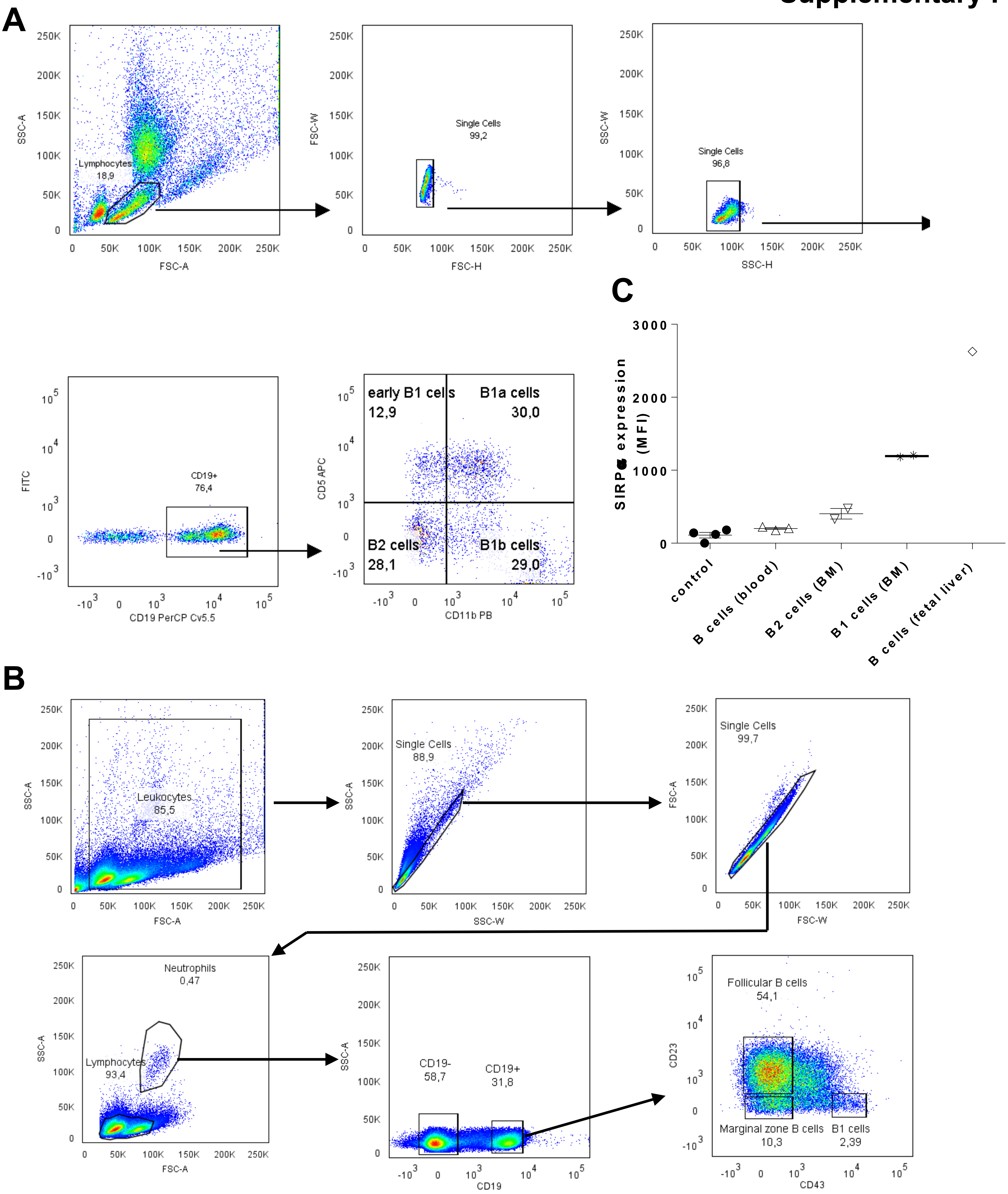

Suppl. Figure 1. Gating strategy for (**A**) peritoneal cavity B1a cells ($B220^{+}/CD19^{+}CD5^{+}CD11b^{+}$) and B1b ($B220^{+}/CD19^{+}CD5^{-}CD11b^{+}$) cells and (**B**) splenic B cell subsets: B1 cells ($B220^{+}/CD19^{+}CD43^{hi}CD23^{-}$), marginal zone B cells ($B220^{+}/CD19^{+}CD43^{-}CD23^{-}$) and follicular B cells ($B220^{+}/CD19^{+}CD43^{-}CD23^{+}$) shown in main Fig. 1B,C. **C**, SIRPα expression on blood, fetal liver, and bone marrow (BM) B cells.

Supplementary Fig. 2

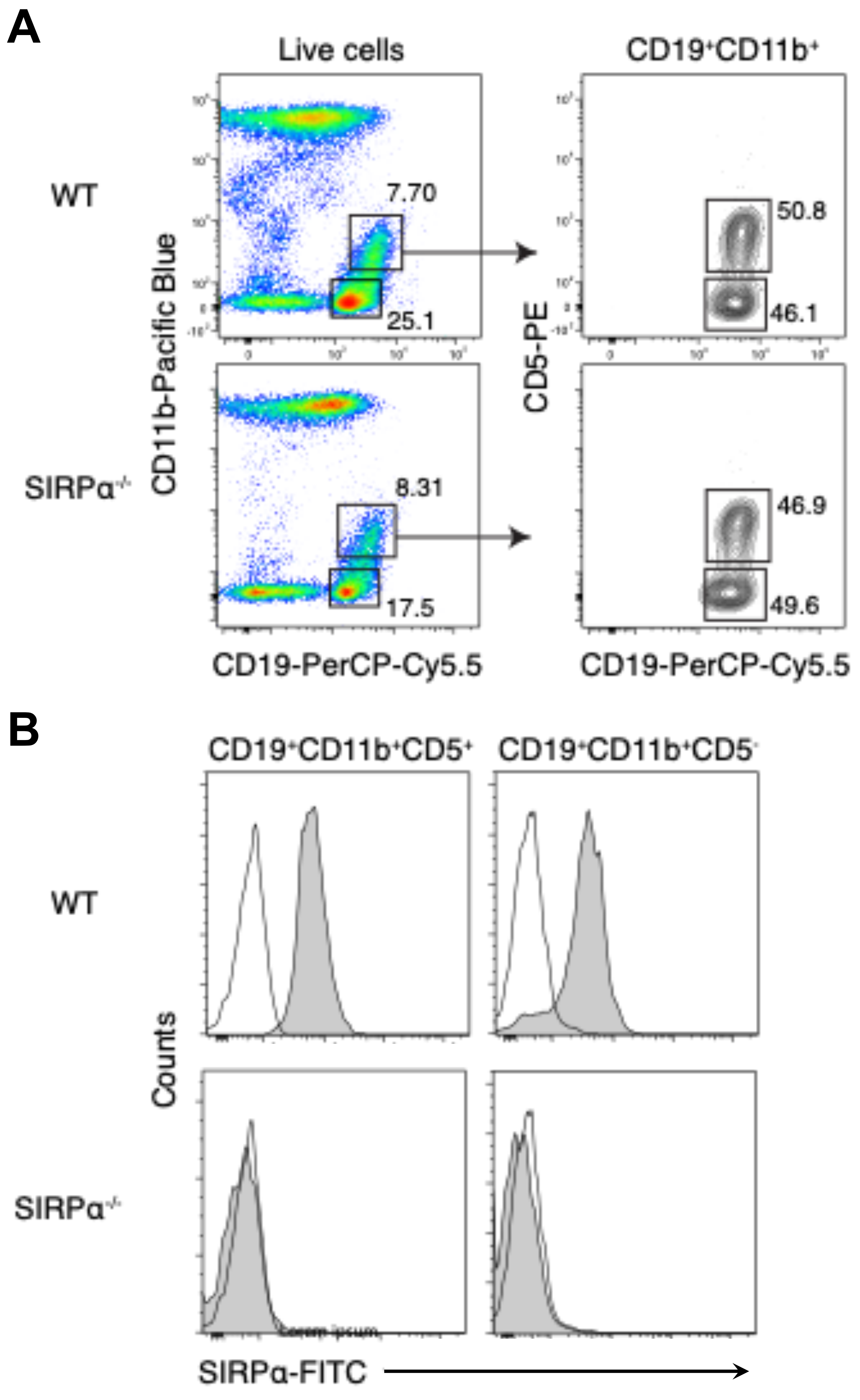

Suppl. Figure 2. Lack of staining for SIRPa on peritoneal B1 cells from SIRPα-/- mice. **A**, gating strategy for the identification of $CD19^+CD11b^+CD5^+$ B1a and $CD19^+CD11b^+CD5^-$ B1b cells from WT and SIRPα$^{-/-}$ mice. **B**, absence of staining for SIRPα (using FITC-conjugated p84 mAb) on peritoneal B1a and B1b cells in SIRPα$^{-/-}$ mice.

Supplementary Fig. 3

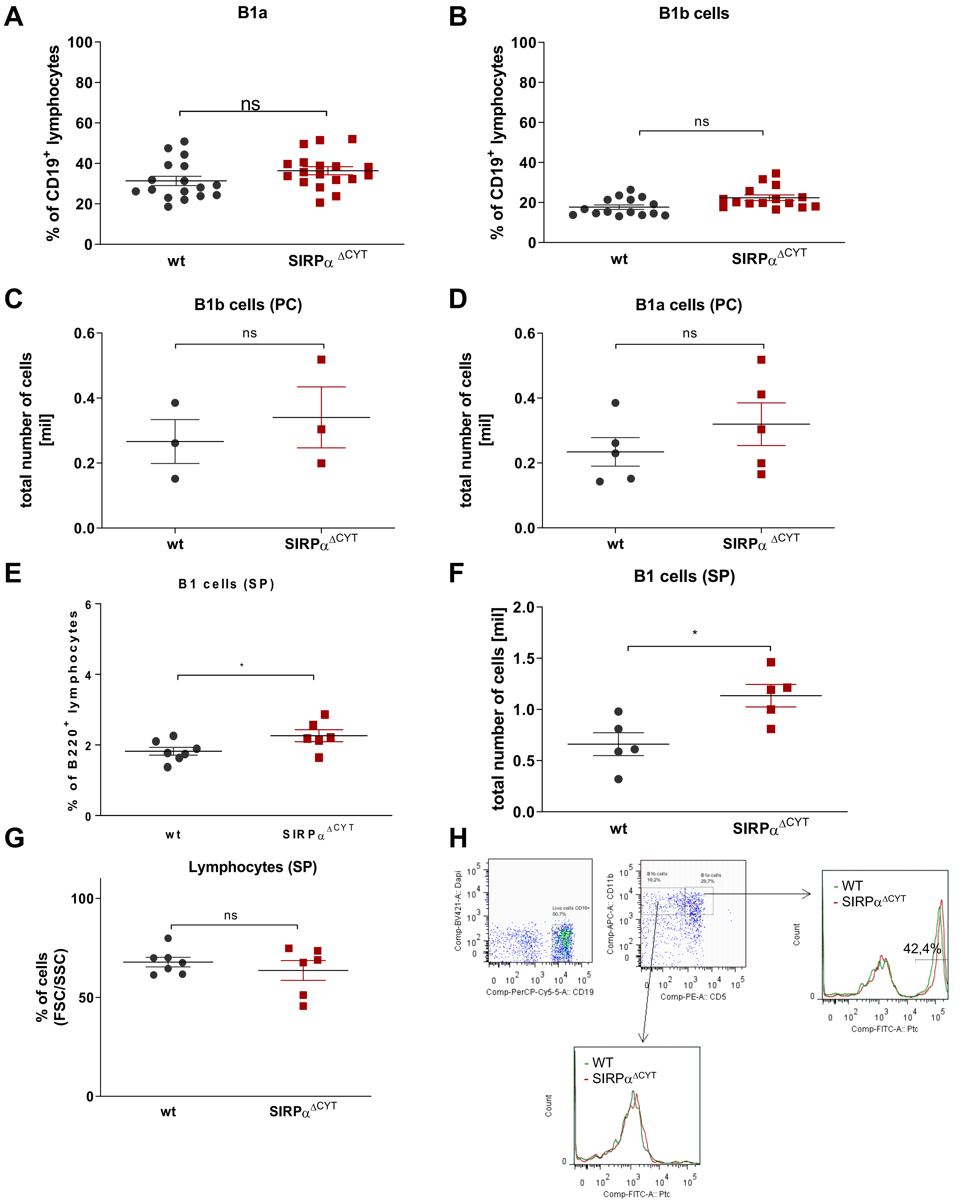

Suppl. Figure 3. SIRPα$^{\Delta CYT}$ mice have enhanced splenic B1 cell numbers (suppl. data to main Table 1and Fig. 3A) **A-D,** Percentages and the absolute numbers of peritoneal cavity (PC) B cells. **E, F,** Percentages and the absolute numbers of splenic (SP) B1 cells. **G,** Percentages of splenic lymphocytes. **H**, Binding of phosphatidylcholine (PtC) to PC B1a and B1b cells. Statistical analysis was performed by unpaired Student t-test, *p<0.05.

Supplementary Fig. 4

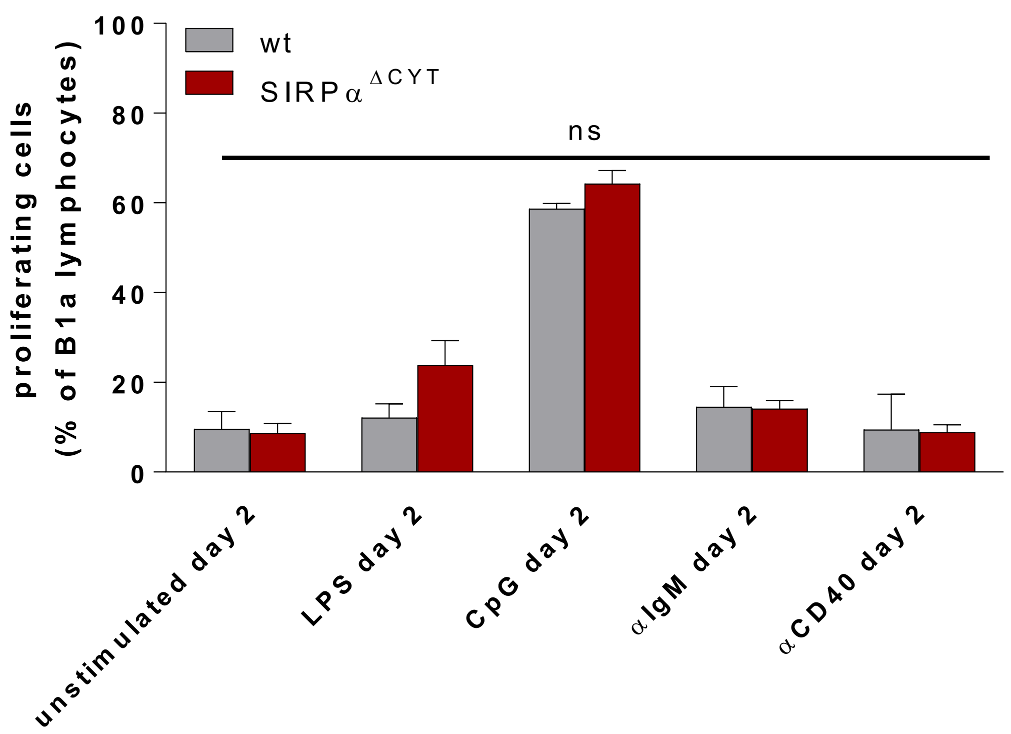

Suppl. Fig. 4. B cells were isolated from the peritoneal cavity of either wt or SIRPα$^{\Delta CYT}$ mice and either left unstimulated or incubated with indicated stimuli for 48h after labeling with CFSE dye. Dilution of the dye after cell division was determined by flow cytometry on B1a cells (gated for CD19+, CD5+, CD11b+ lymphocytes) and percentage of proliferating cells was calculated. Data are presented as mean ±SEM and are representative of 2 (wt) and 3 (SIRP$^{\alpha\Delta CYT}$) individual mice. Statistical analysis was performed by unpaired Student t-test, corrected for multiple comparisons with Holm-Sedak method where applicable; ns, for none of the conditions there was a statistically significant difference ($p<0.05$).

Supplementary Fig. 5

A

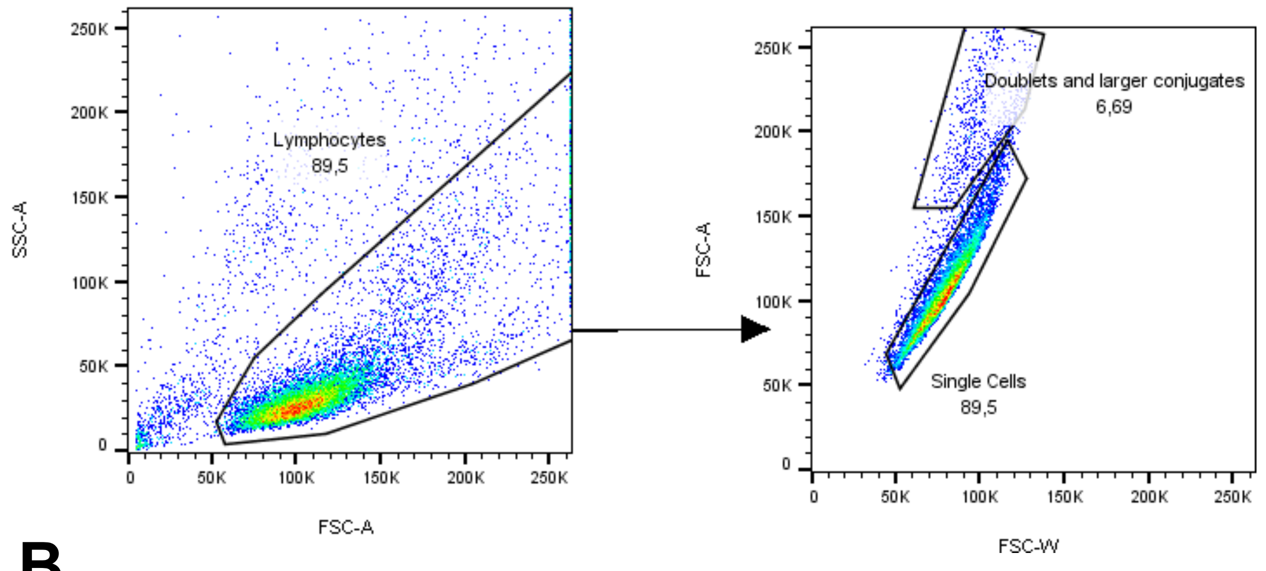

B

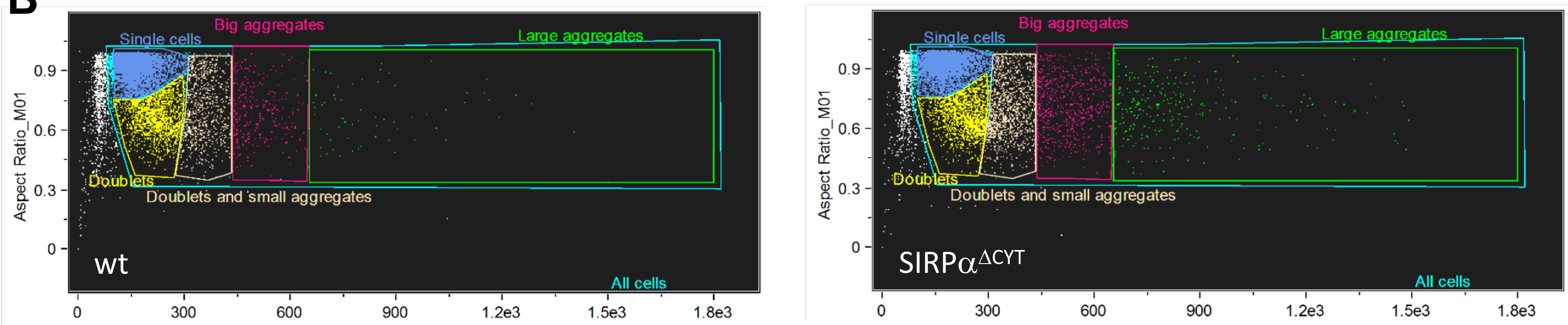

wt	Count	% Gated
All	10810	100
All cells	9844	91,1
Single cells	6644	61,5
Doublets	1651	15,3
Small agregates	761	7,04
Medium aggregates	267	2,47
Large aggregates	63	0,58

SIRPα$^{\Delta CYT}$	Count	% Gated
All	11271	100
All cells	10330	91,7
Single cells	6316	56
Doublets	1547	13,7
Small agregates	1158	10,3
Medium aggregates	568	5,04
Large aggregates	295	2,62

C

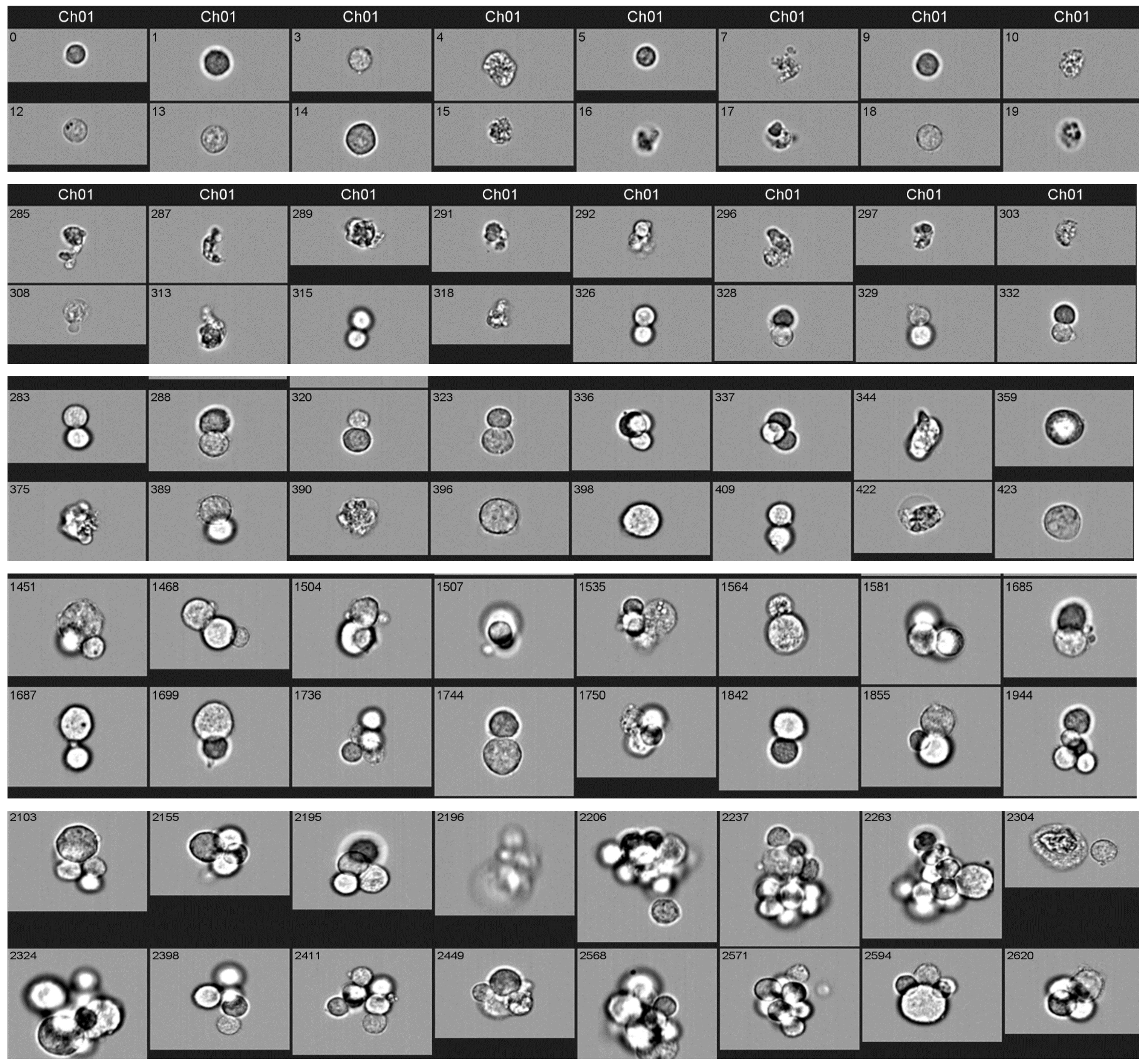

Single cells

Doublets

Small aggregates

Medium aggregates

Large aggregates

Suppl. Fig. 4. A, Sorted B1a cells cultured with LPS showing the formation of conjugates detected by regular flow cytometry. **B,** Imaging flow cytometry for analyzing the extent of conjugation of B1a cells (representative examples shown). **C** Random images of conjugates from the corresponding gates shown in panel B.

Supplementary Fig. 6

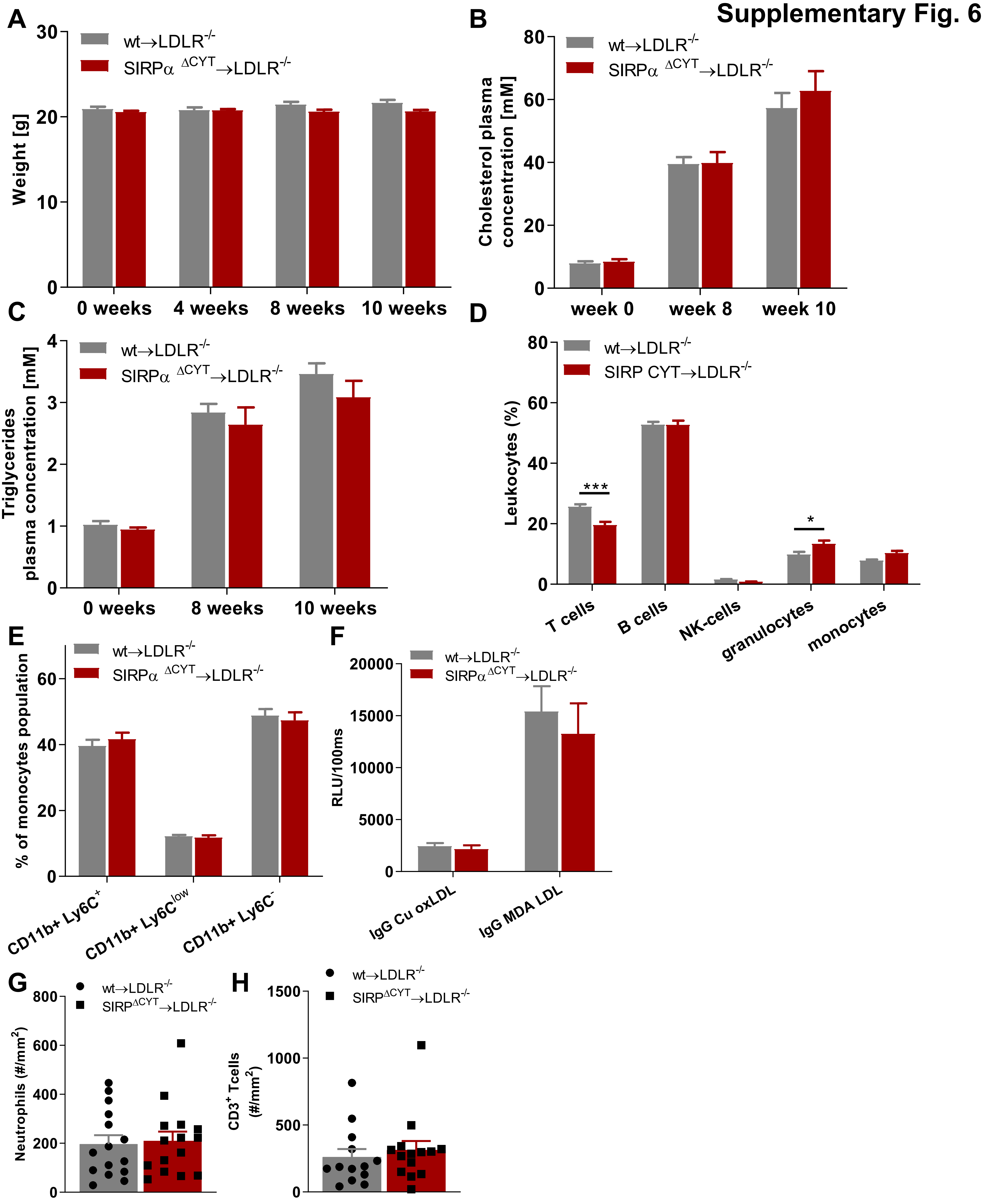

Suppl. Figure 5. Lack of SIRPα signaling protects mice from atherosclerosis (supplementary data to main Fig. 4). LDLR-/- mice reconstituted with either wt or SIRPα$^{\Delta CYT}$ bone marrow show comparable weight (A), plasma levels of cholesterol (B) and triglycerides (C), blood leukocyte composition (D), including the indicated monocyte subsets (E), and plasma IgG antibodies against modified LDL, including Cu oxLDL and MDA LDL (F), and atherosclerotic plaque neutrophil (G) and T cell (H) composition during (A-C) and at the end (i.e. 10 weeks; D-H) of the high fat diet period. Statistical analysis was performed by unpaired Student t-test, *p<0.05, ***p<0.001, other values non-significant.
